# DC Priming by *M. vaccae* Inhibits Th2 Responses in Contrast to Specific TLR2 Priming and Is Associated with Selective Activation of the CREB Pathway

**DOI:** 10.1371/journal.pone.0018346

**Published:** 2011-04-01

**Authors:** Nina Le Bert, Benjamin M. Chain, Graham Rook, Mahdad Noursadeghi

**Affiliations:** Infection and Immunity, University College London, London, United Kingdom; University Hospital Freiburg, Germany

## Abstract

The environmental mycobacterium, *M. vaccae* has been used in mouse models to support the contemporary hygiene hypothesis that non-pathogenic microorganisms reduce allergy associated T helper (Th)2 responses and inflammatory diseases by augmenting regulatory T cells. However, data for human models and possible mechanisms are limited. We tested the effect of innate immune interactions between human DC and *M. vaccae* on DC-dependent T cell responses. *M. vaccae* activation of DC via Toll like receptor (TLR)2 was compared to a specific TLR2 ligand (Pam_3_CSK4) and alternative stimulation with a TLR4 ligand (LPS). *M. vaccae* induced DC dependent inhibition of Th2 responses, in contrast to Pam_3_CSK4, which had the opposite effect and LPS, which had no polarizing effect. DC maturation, gene expression and cytokine production, in response to each stimulus did not correlate with the specific functional effects. Comparable DC transcriptional responses to *M. vaccae* and Pam_3_CSK4 suggested that TLR2 mediated transcriptional regulation was not sufficient for inhibition of Th2 responses. Transcription factor enrichment analysis and assessment of signaling events, implicated a role for selective early activation of the CREB pathway by *M. vaccae*. Further study of the CREB pathway may provide novel insight into the molecular mechanisms of DC-dependent T cell polarization.

## Introduction

The role of dendritic cells (DC) in shaping adaptive immune responses has been subject to extensive research with the aim of therapeutic modulation of the immune system [1], [2]. The hygiene hypothesis suggests that non-pathogenic or commensal microorganisms may influence the nature of adaptive immunity [3], [4]. In animal models of asthma and eczma [5], [6], [7], [8], [9], [10] administration of heat-killed preparations of *M. vaccae* reduce antigen-specific allergic responses. A number of human clinical trials showed that *M. vaccae* may also have therapeutic effects in asthma or atopic dermatitis [11], [12], albeit inconsistently [13], [14], [15]. In addition, *M. vaccae* might enhance host defenses against tuberculosis (TB) [16], [17], [18]. Data from animal models suggest that *M. vaccae* exerts these effects by reducing allergy-associated T helper (Th)2 responses, by increasing regulatory T cell (Treg) responses [6], and by increasing cell-mediated immunity-associated Th1 responses [19]. Whether these effects are also evident in human cellular immunology and the underlying mechanisms are not known. DC support Th cell responses through antigen presentation and provision of co-stimulatory signals [20]. In view of their potency to activate naive T cells, DC-T cell interactions are thought to influence Th polarization through changes in the cytokine microenvironment [1], [21] and by the strength of TCR stimulation [22], [23], [24], but the molecular mechanisms are not established. Microbial organisms interact with DC through innate immune receptors and consequently stimulate intracellular signals that lead to genome-wide transcriptional changes, expression of cell surface molecules and secretion of cytokines and chemokines, which contribute to DC-T cell interactions [1] and may contribute to differential polarization of Th cells. Such effects have been reported for DC primed with *Bordetella pertussis* to promote mixed Th1/Th17 polarization, DC primed with schistosomal omega-1 protein that induced Th2 cells, or with probiotics that increased Treg responses [22], [25], [26]. In a mouse model of ovalbumin-induced airway allergy, *M. vaccae* induced inhibition of Th2 responses together with the development of CD11c^+ve^ cells, possibly DC, associated with increased expression of immunomodulatory cytokines [27]. We tested the hypothesis that *M. vaccae* induces changes to human Th polarized responses that are mediated by DC. We used heat-killed *M. vaccae* similar to preparations used in the animal and human trials. By qualitative comparison of DC responses to *M. vaccae* and to other stimuli that use common or different innate immune receptors, we sought to obtain new insights into the mechanisms by which differential innate immune activation of DC control Th polarization. We found that genome-wide transcriptional responses to *M. vaccae* are directly comparable to specific Toll-like receptor (TLR)2 stimulation, but associated with divergent effects on DC-dependent Th2 responses. By focusing on specific transcriptional responses to each stimulus, we identified and confirmed selective early activation of the CREB pathway by *M. vaccae*. Further assessment of upstream and downstream signaling events may lead to better resolution of the molecular mechanisms by which DC control polarization of Th responses.

## Results

### *M. vaccae* induces dose dependent maturation of monocyte derived dendritic cells and can stimulate TLR2 dependent cellular activation

**Figure 1:**
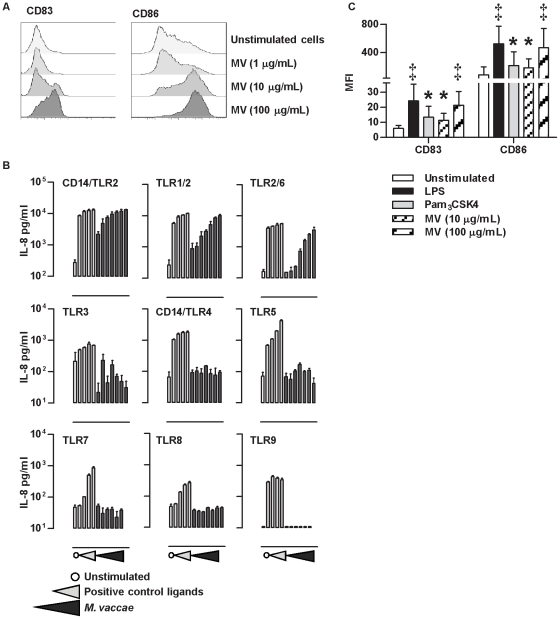
Maturation of DC by LPS, Pam3CSK4 and M. vaccae. Cell surface CD83 and CD86 expression in DC showed dose dependent response to 24 hours stimulation with *M. vaccae* (**A**). Flow cytometry histograms show representative data from multiple experimental replicates. (**B**) HEK293 cells stably transfected with plasmids expressing the TLR receptors ±CD14 as indicated, were treated with medium (**○**), increasing concentrations of positive control ligands or *M. vaccae*. TLR2/CD14: heat killed *Listeria monocytogenesis* (10^7^–10^8^/mL), TLR1/2: Pam_3_CSK4 (62.5–500 ng/mL), TLR2/6: FSL-1 (25–200 ng/mL), TLR3: poly(I:C) (25–200 µg/mL), TLR4/CD14: LPS (0.1–100 ng/mL), TLR5: Flagellin (0.5–4 µg/mL), TLR7: Gardiquimod (0.625–5 µg/mL), TLR8: CL075 (5–40 µg/mL), TLR9: OND2006 (0.3–2.5 µM) and *M. vaccae* (1–1000 µg/mL). TLR-dependent cellular activation was measured after 24 hours by ELISA quantifying IL-8 concentrations in the cell culture supernatants. Data show mean (±SD) of three independent experiments. (**C**) Upregulation of CD83 and CD86 expression (mean fluorescence intensity) in response to *M. vaccae* (100 µg/mL) was comparable to that of LPS (100 ng/mL) and significantly greater than the response to Pam_3_CSK4 (1 µg/mL). Stimulation of DC with 10-fold lower concentration of *M. vaccae* (10 µg/mL) generated comparable DC maturation to Pam_3_CSK4. Bars represent mean ±SD of 14 separate experiments (^★^ denotes significant differences to unstimulated cells,and ^‡^ denotes significant differences to stimulation with Pam_3_CSK4 or *M. vaccae* (10 µg/mL), p<0.001 by paired t tests).

**Figure 2:**
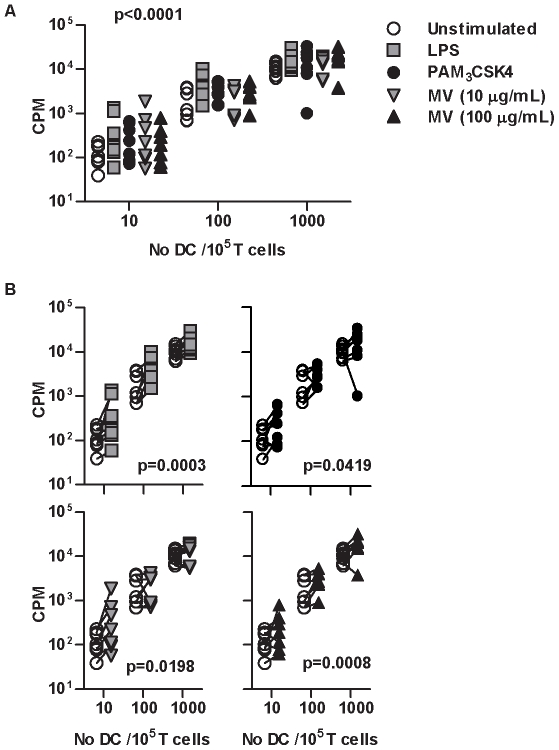
Priming of DC with M. vaccae enhances allogeneic T cell proliferation. Naive CD4^+^ T cell proliferation was assessed by thymidine incorporation (CPM) after 3 days stimulation with allogeneic DC. The effects of DC number and DC priming with the stimuli indicated were assessed by repeated measure 2-way ANOVA. Increasing DC∶T cell ratios were associated with significantly increased T cell proliferation (**A**), and in comparison to unstimulated cells, DC primed with each of the stimuli also significantly increased proliferation (**B**). There were no significant differences between the stimuli. Each experiment is represented by paired data points.

The hallmark of innate immune DC priming for T cell activation is upregulated expression of co-stimulatory molecules such as CD86 and the maturation marker CD83 [2]. *M. vaccae* stimulates dose dependent maturation of DC in this way (Figure 1A), at concentrations that are comparable to those achieved by intradermal injection of 1 mg in clinical trials. In order to develop insight into the specific consequences of DC priming by *M. vaccae*, we sought to make comparisons with other stimuli for cellular receptors which are shared or distinct from those of *M. vaccae*. Screening of reporter cell lines expressing different TLR homo- or heterodimer combinations (Figure 1B) confirmed TLR2 dependent gene expression in response to *M. vaccae*, in keeping with existing literature on TLR2 interactions with mycobacteria [28]. The lack of TLR4 stimulation confirmed the absence of lipopolysaccharide (LPS) contamination in this preparation, and allowed us to compare the effects of *M. vaccae* on DC, to TLR4 stimulation with LPS and specific TLR2 stimulation with Pam_3_CSK4. Comparison of maximal increases in CD83 and CD86 expression, suggested that LPS and *M. vaccae*-induced maturation was significantly greater than that of Pam_3_CSK4 (Figure 1C). Therefore a 10-fold lower concentration of *M. vaccae* (10 µg/mL), to induce comparable maturation to Pam_3_CSK4 was also included in the experimental paradigm. Next we tested the effect of priming DC with each of these stimuli, 24 hours before addition of naive allogeneic T cells, thereby excluding memory T cells for mycobacteria (Figure 2A). DC number and innate immune priming were independently associated with T cell proliferation. This effect was statistically more significant in DC primed with LPS or 100 µg/mL *M. vaccae* in comparison to Pam_3_CSK4 or 10 µg/mL *M. vaccae* (Figure 2B).

### *M. vaccae* attenuates Th2 responses

**Figure 3:**
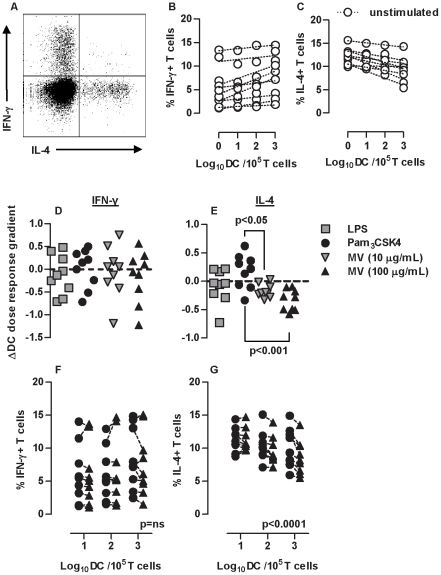
Stimulation of DC with M. vaccae attenuates Th2 responses. In 3 day allogeneic co-cultures of DC with naive CD4^+^ T cells, IFN-γ^+^ and IL-4^+^ producing T cells were enumerated by intracellular immunofluorescence staining and flow cytometry, after PMA/ionomycin stimulation (**A**). Increasing DC∶T cell ratios were associated with increased proportions of IFN-γ^+^ cells, but decreased proportions of IL-4^+^ cells (p<0.0001, 2-way repeated measure ANOVA) (**B–C**). In order to assess the effect of DC priming in this model, the regression relationship between DC∶T cell ratio and proportions of IFN-γ^+^ or IL-4^+^ cells was determined for each experiment (dotted lines) and the gradient of these relationships in unprimed DC were compared to those of primed DC (**D–E**). DC priming had no significant effect on DC-dependent IFN-γ polarization of T cells, priming with *M. vaccae* significantly enhanced DC-dependent reduction of IL-4^+^ producing T cells in contrast to priming with Pam_3_CSK4, which had the opposite effect (paired t test). Direct comparison, showed significant reduction (2-way repeated measure ANOVA) of IL-4^+^ T cells with increasing numbers of DC primed with *M. vaccae* compared to those primed with Pam_3_CSK4 (**G**). No differential effects on IFN-γ^+^ T cells were evident (**F**). Data points represent results from individual experiments.

**Figure 4:**
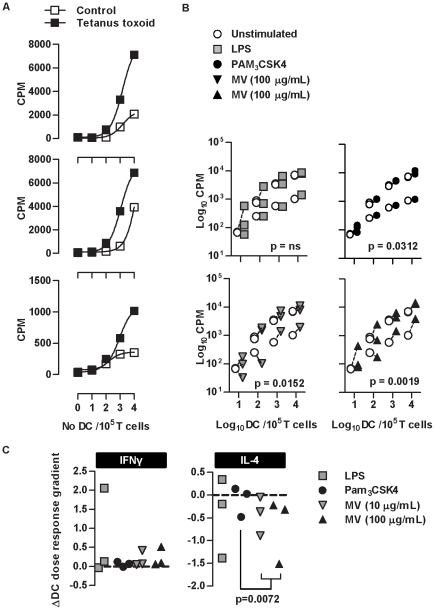
Stimulation of MDDC with M. vaccae attenuates Th2 differentiation in antigen specific responses. T cell proliferation stimulated by 3 day culture with autologous DC primed for 24 hours ± tetanus toxoid (TT) showed DC and TT-dependent responses in cells from 3 separate donors (**A**). Repeated measure 2-way ANOVA showed a significant increase in proliferative responses associated with the priming of MDDC with Pam_3_CSK4 and *M. vaccae* (**B**). Data points represent individual experiments and lines link paired data from the same donor/experiment. In antigen-specific responses to tetanus toxoid, in order to assess the effect of DC priming on Th polarization, the regression relationship between DC∶T cell ratio and proportions of IFN-γ^+^ or IL-4^+^ cells was determined after 3 days and the gradient of these relationships in unprimed DC compared to those of primed DC (**C**). DC priming had no significant effect on DC-dependent IFN-γ polarization of T cells, but priming with *M. vaccae* significantly enhanced DC-dependent reduction of IL-4^+^ producing T cells in comparison to priming with Pam_3_CSK4 (paired t test).

We then tested the qualitative effect of DC priming with each stimulus on allogeneic T cell responses by intracellular staining for IFNγ and IL-4, as markers for Th1 and Th2 responses respectively. We found no double positive cells in these experiments (Figure 3A). Increasing numbers of DC showed a positive correlation with Th1 and negative correlation with Th2 responses (Figure 3B–C). We therefore tested the effect of innate immune priming across the range of DC∶T cell ratios. LPS, Pam_3_CSK4 and *M. vaccae* stimulation of DC did not affect the relationship between DC and Th1 responses (Figure 3D,F). However, Pam_3_CSK4 priming of DC was associated with sustained Th2 responses, reducing the inverse relationship between number of DC and proportion of IL-4^+ve^ T cells, and DC priming with *M. vaccae* augmented this negative relationship (Figure 3E,G). This is clearly shown in pair wise comparisons of the effect of DC priming with Pam_3_CSK4 and *M. vaccae*, on IFNγ or IL-4 producing cells (Figure 3F–G). *M. vaccae* priming of DC was associated with greater reduction of Th2 responses with increasing number of DC (Figure 3G), emphasizing the role of DC. The magnitude of this effect was similar to that of innate immune priming of DC on T cell proliferation (Figure 2). The same effects were also evident in antigen (tetanus toxoid) specific responses by memory T cells (Figure 4A–C). Greater inhibition of IL-4 producing T cells by 100 µg/mL *M. vaccae* compared to 10 µg/mL *M. vaccae* suggested a dose-response relationship for this effect (Figure 3E). Significant differences between 10 µg/mL *M. vaccae* and Pam_3_CSK4 priming of DC were also evident despite comparable levels of DC maturation. In addition, LPS priming of DC did not significantly attenuate Th2 responses despite inducing similar levels of DC maturation to priming with 100 µg/mL *M. vaccae*. Taken together, these findings show that enhanced DC-dependent inhibition of Th2 responses were specific to priming with *M. vaccae* and independent of levels of DC maturation.

**Figure 5:**
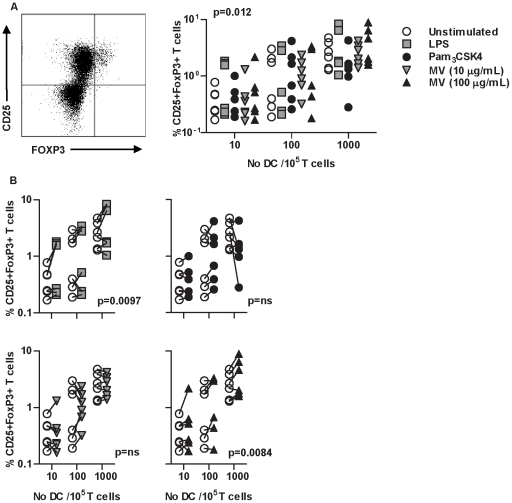
Priming of DC with M. vaccae induces CD25+FoxP3+ T cells. In 6 day allogeneic cocultures of DC with naive CD4^+^ T cells, CD25^+^ FoxP3^+^ T cells were enumerated by intracellular immunofluorescence staining and flow cytometry. The effects of DC number and DC priming with the stimuli indicated were assessed by repeated measure ANOVA. Increasing DC∶T cell ratios were associated with increased proportions of CD25^+^FoxP3^+^ T cells (**A**). In comparison to unstimulated cells, DC primed with LPS or *M. vaccae* (100 µg/mL) also significantly increased CD25^+^FoxP3^+^ T cells (**B**). Each experiment is represented by paired data points.

Previous reports from animal models suggested that inhibition of Th2 responses may be the result of enhanced Treg responses in mice receiving *M. vaccae*. In the present model, there was a clear relationship between the number of DC and induction of CD25^high^/FoxP3^high^ cells (Figure 5A). Priming of DC with LPS or 100 µg/mL *M. vaccae* significantly enhanced this induction, but this effect was not evident with Pam_3_CSK4 or 10 µg/mL *M. vaccae* (Figure 5B). These findings did not demonstrate a consistent correlation with effects of DC priming on Th2 responses or inhibition of T cell proliferation. In addition we found no evidence of IL-10 production by these cells using intracellular cytokine staining or ELISA of cell culture supernatants (data not shown). Therefore the CD25^high^/FoxP3^high^ phenotype may be a feature of T cell activation rather than Treg differentiation and was not investigated further in the present study.

### The predominant transcriptional and cytokine responses to *M. vaccae* and specific TLR2 stimulation are comparable

**Figure 6:**
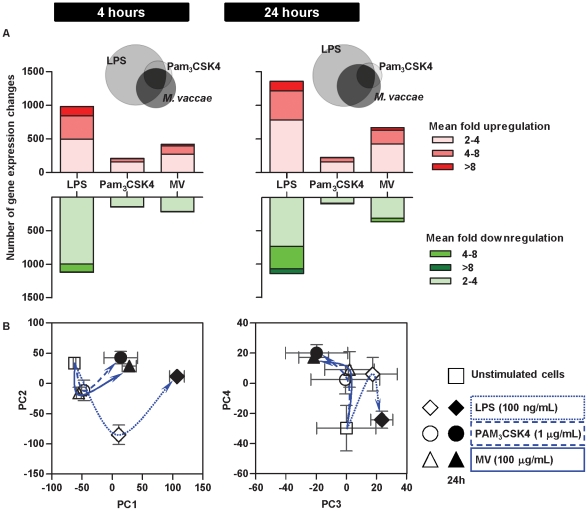
The predominant transcriptional responses to M. vaccae and specific TLR2 stimulation are comparable. (**A**) Quantitative comparison of (>2-fold) up- and downregulated gene expression changes in DC after 4 h of stimulation with LPS (100 ng/ml), Pam_3_CSK4 (1 µg/ml) or *M. vaccae* (100 µg/ml) and qualitative Venn diagram comparison of (>2-fold) upregulated genes. Data are derived from the mean of three separate experiments using cDNA microarray gene expression profiling. (**B**) Principal component analysis (PCA) of transcriptional profiling differences in DC stimulated for 4 h or 24 h with LPS (100 ng/ml), Pam_3_CSK4 (1 µg/ml) or *M. vaccae* (100 µg/ml) and control unstimulated DC. Data points show mean (±SEM) PCA scores for three independent experiments. Lines and arrows indicate vector of transcriptional responses to each stimulus with time. PC1 and PC2 show common transcriptional changes to all stimuli, which are quantitatively greatest as a result of LPS stimulation. PC3 and PC4 show divergent responses in LPS stimulated cells compared to Pam_3_CSK4 or *M. vaccae*. Transcriptional profiles in *M. vaccae* and Pam_3_CSK4 stimulated cells are closely aligned in each component.

**Figure 7:**
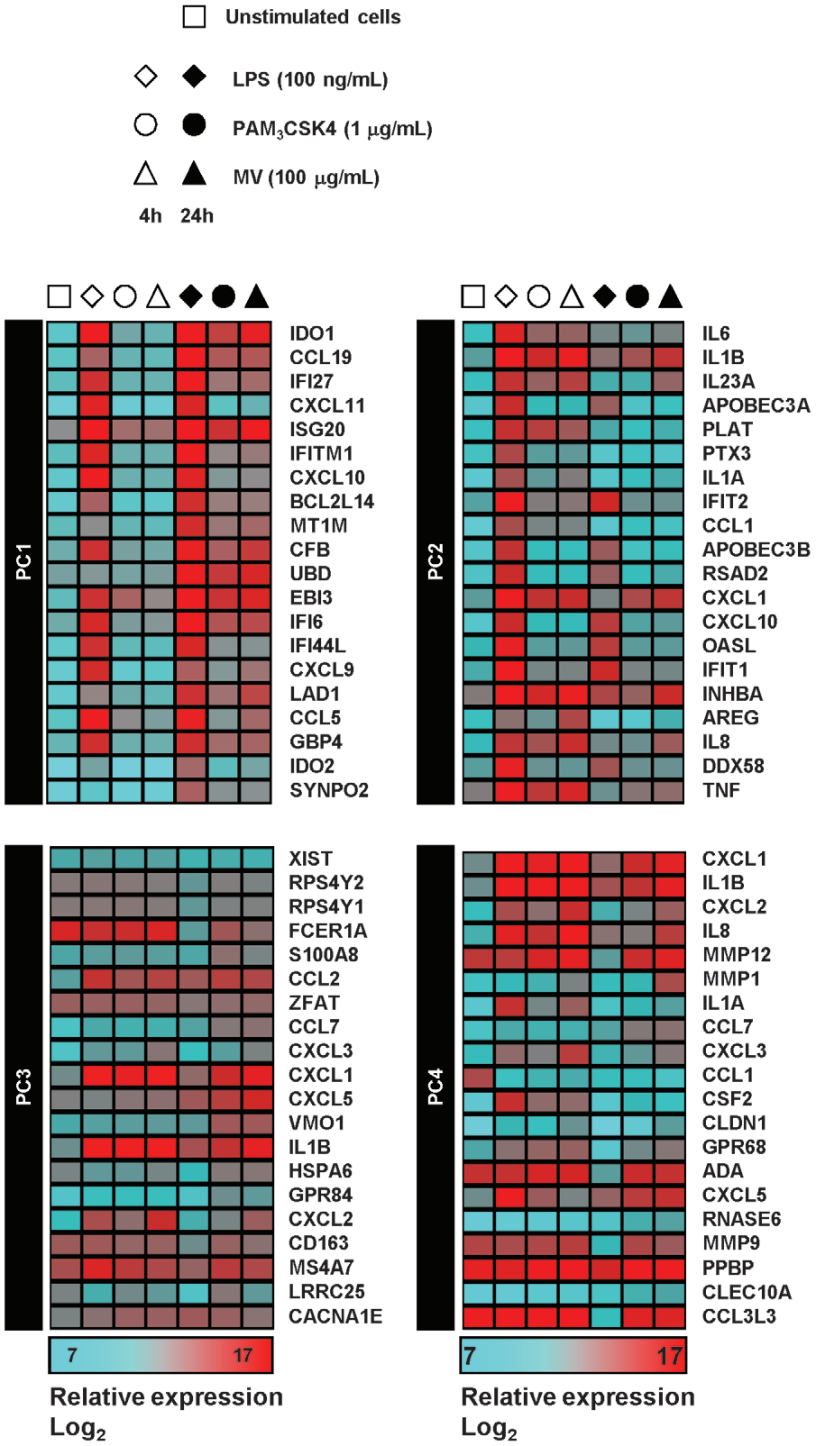
Relative expression levels for gene expression differences in differentially stimulated DC. Heat map representation of relative gene expression levels for top 20 genes which are responsible for the greatest variance in the first four principle components (PC) of gene expression differences in differentially stimulated DC. Data are derived from the mean of three separate experiments using cDNA microarray gene expression profiling.

In order to investigate the differential effects of *M. vaccae* and Pam_3_CSK4 on DC-mediated inhibition of Th2 responses, we next compared genome-wide transcriptional responses in DC primed with LPS, Pam_3_CSK4 and 100 µg/mL *M. vaccae*. The frequency distribution of significantly (>2-fold) upregulated and downregulated genes suggested that LPS had the greatest effect on gene expression, followed by *M. vaccae* and then Pam_3_CSK4 (Figure 6A). In addition, qualitative comparison of gene expression changes suggested shared and stimulus specific responses (Figure 6A), but this may simply reflect differences in many genes which were only modestly affected. We therefore used principle component analysis (PCA) of transcriptional profiles to compare components of the data that are responsible for the greatest gene expression differences (Figure 6B). In this analysis, LPS stimulation of DC induced the greatest gene expression changes in principle component (PC)1 and PC2. Gene expression changes represented by PC2 at 4 hours returned to baseline levels at 24 hours and gene expression changes represented by PC1 at 4 hours increased further at 24 hours. In these components, gene expression profiles in DC primed with Pam_3_CSK4 or *M. vaccae*, showed the same pattern of responses, albeit quantitatively less than responses to LPS. PC3 and PC4 showed a different pattern of gene expression changes in stimulated DC. In PC3, LPS stimulation caused transcriptional changes at 4 hours and 24 hours that were divergent to those of DC stimulated with Pam_3_CSK4 or *M. vaccae*. PC4 showed comparable transcriptional changes associated with all three stimuli at 4 hours, but divergent responses at 24 hours. Quantitative, qualitative and time course assessment of genome-wide transcriptional responses by PCA, suggested that the major transcriptional responses in Pam_3_CSK4 and *M. vaccae* stimulated DC were comparable. This was reflected in gene expression data for the top 20 genes that make the greatest contribution to each PC (Figure 7).

**Figure 8:**
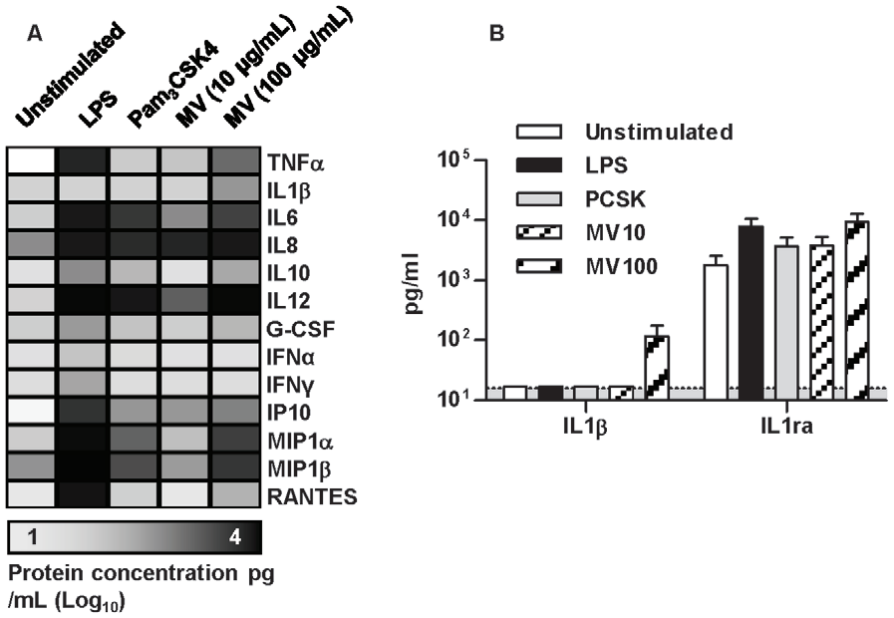
responses to M. vaccae and specific TLR2 stimulation are comparable at protein level. (**A**). Selected cytokine and chemokine levels in the supernatants of DC stimulated for 24 hours with LPS, Pam_3_CSK4 or *M. vaccae* (MV) and control unstimulated DC are presented in a heat map showing mean of three experiments. In general, this showed highest levels in LPS stimulated cells and comparable levels in Pam_3_CSK4 and *M. vaccae* stimulated cells. Concentrations of IL-1β and IL-1 receptor antagonist (ra) are shown in (**B**). Data show mean (±SD) of three independent experiments.

Upregulated genes in stimulated DC were significantly enriched for extracellular factors with cytokine and chemokine activity (Table 1). Therefore to validate the expression profiling analysis and look for discordance between transcriptional and protein responses, we measured cytokine release by differentially stimulated DC (Figure 8). In keeping with the microarray data, LPS stimulated the largest responses. Pam_3_CSK4 and *M. vaccae* induced comparable smaller responses. Interestingly, although increased gene expression of IL-1β was induced by all these stimuli, the protein was only detected at modest concentrations in DC stimulated with 100 µg/mL *M. vaccae*, suggesting activation of the inflammasome pathway [29]. We considered the possibility that IL-1β may contribute to DC-dependent co-stimulation of T cells that is responsible for the inhibition of Th2 responses associated with *M. vaccae* priming. However, the homeostatic regulator of IL-1β activity, IL-1-receptor antagonist (ra) was also present in cell culture supernatants at high concentrations that were likely to negate any biological activity of relatively small increase in IL-1β concentration (Figure 8B). In summary, the major genome-wide transcriptional responses in DC stimulated with *M. vaccae* were reproduced by specific TLR2 stimulation, but these stimuli had divergent effects on DC-dependent Th cell polarization. These findings strongly suggested that the programme of transcriptional responses to *M. vaccae* in general and those mediated by TLR2 specifically were insufficient to inhibit DC-dependent Th2 responses.

**Table 1:** Gene ontology terms from top 3 enriched groups of genes identified by functional annotation clustering analysis of genes that show >2-fold upregulation in DC stimulated with *M. vaccae* for 4 hours.

Gene ontology term	P value	No of genes	% of gene list	Fold enrichment
GO:0005125∼cytokine activity	3.25^−20^	35	8.71	7.54
GO:0009611∼response to wounding	4.85^−19^	56	13.93	4.06
GO:0012501∼programmed cell death	3.88^−16^	56	13.93	3.52
GO:0016265∼death	5.97^−16^	61	15.17	3.24
GO:0006915∼apoptosis	8.41^−16^	55	13.68	3.51
GO:0008219∼cell death	1.82^−15^	60	14.93	3.21
GO:0006952∼defense response	8.22^−15^	54	13.43	3.37
GO:0006954∼inflammatory response	2.98^−14^	38	9.45	4.49
GO:0005615∼extracellular space	1.19^−12^	49	12.19	3.19
GO:0044421∼extracellular region part	6.35^−12^	58	14.43	2.69
GO:0005576∼extracellular region	2.04^−05^	73	18.16	1.62

### *M. vaccae* selectively stimulates early activation of the CREB pathway

**Figure 9:**
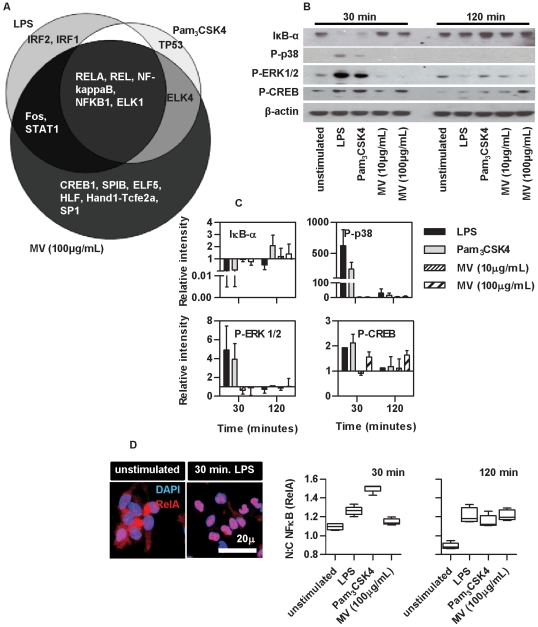
M. vaccae selectively stimulates early activation of the CREB pathway. Analysis of significantly enriched transcription factor binding sites in genes upregulated by DC stimulation (4 h) is shown (**A**). Western blot analysis of candidate innate immune signaling events in DC, show comparable degradation of IκBα, and phosphorylation of p38, ERK1/2 and CREB in DC after 30 min and 120 min of stimulation with LPS (100 ng/ml) and Pam_3_CSK4 (1 µg/ml), but selective activation of the CREB pathway by *M. vaccae* (**B**). Quantitative densitometry data from 3 separate experiments are shown in (**C**). Bars represent mean ±SD. Quantitative confocal immunofluorescence staining used to detect NFκB RelA (p65) nuclear translocation in response to innate immune stimulation of DC (**D**) showed that activation of the classical NFκB pathway was evident by 30 minutes in response to LPS or Pam_3_CSK4, and by 120 minutes in response to *M. vaccae* (**B**). Representative images from 3 separate experiments are shown. Box and whisker plots represent median, and range of data from approximately 500 single cell measurements.

We next focused on the differences in *M. vaccae* and Pam_3_CSK4 induced transcriptional responses to assess alternative innate immune signaling pathways, which may contribute to the differential functional effects under study. Each combination of shared or exclusive gene lists upregulated by LPS, Pam_3_CSK4 or *M. vaccae* was assessed for statistical enrichment of transcription factor binding sites (Figure 9A). As expected, this showed enrichment for NFκB components in the common response for all stimuli. The most highly enriched (Z-score 19.98) transcription factor in genes exclusively upregulated by *M. vaccae* was found to be cyclic AMP responsive element binding protein (CREB)1. This finding was supported by assessment of signaling events in differentially stimulated DC to assess IκBα degradation in the classical NFκB pathway, phosphorylation of p38 and ERK1/2 in the MAP kinase pathway and phosphorylation of CREB1. Despite marked differences in DC maturation, transcriptional and cytokine responses in DC stimulated with LPS and Pam_3_CSK4, the innate immune signaling events assessed here showed a very similar profile (Figure 9B–C) with evidence for activation in all of the pathways tested. However, *M. vaccae* induced selective early and sustained activation of the CREB pathway. The transcriptional profiling data suggested that *M. vaccae* also activates NFκB pathways, but the time course of NFκB RelA nuclear translocation in response to *M. vaccae* was slower in comparison to LPS or PCSK stimulation (Figure 9D). These data suggest that investigation of the pathway upstream of CREB activation may identify distinct innate immune signaling events that are involved in DC-dependent inhibition of Th2 responses.

## Discussion

The power of DC control over T cell function is self-evident in our experimental model. The major effects on T cell proliferation were directly proportional to the DC∶T cell ratio in both allogeneic and antigen-specific responses, further augmented by innate immune priming of DC that correlated with the magnitude of DC maturation. Increasing DC∶T cell ratio also caused increasing Th1 polarization, potentially as a result of increasing T cell receptor signal strength [23]. In this context, innate immune priming of DC had differential effects on T cell polarization. Our finding that *M. vaccae* priming of DC augmented the DC-dependent reduction of IL-4^+ve^ T cells, suggests that the hypothesis derived from animal models, that *M. vaccae* may reduce allergy by inhibition of Th2 responses [30], [31], may also be operative in human cellular immunology and that this effect is mediated by DC.

We also attempted to address the molecular mechanisms by which DC may inhibit Th2 responses by comparing the effects of *M. vaccae* to those of other stimuli that use common or alternative innate immune cellular activation pathways. Like other mycobacteria, *M. vaccae* can stimulate cells via TLR2 [28], [32], [33] but not TLR4. Therefore, we made comparisons to specific TLR2 and TLR4 stimulation. In stark contrast to the effect of *M. vaccae*, specific TLR2 (Pam_3_CSK4) priming of DC, supported Th2 polarized responses. Others have also shown Pam_3_CSK4 priming of human monocyte derived DC increase Th2 polarization of naive T cells [34]. This is further supported by murine studies showing that administration of a Pam_3_CSK4 with OVA augmented Th2-associated cytokine production by antigen-specific T cells [35]. However conflicting data from mouse allergy models suggest that Pam_3_CSK4 stimulation may increase IFNγ producing Th1 cells [36], [37], cells with a Th1/Treg profile [38], or possibly reduce both Th1 and Th2 cells through CD4^+^ T cell apoptosis [39]. In human blood mononuclear cells from mite sensitized individuals, Pam_3_CSK4 reduced Th2 responses [40], and in whole blood cultures from nematode-infected children Pam_3_CSK4 was shown to have IL-10 inducing capacity [41]. The context specific effects of TLR2 stimulation on T cell responses therefore require further study.

*M. vaccae* may induce cellular activation of DC via receptors such as DC-SIGN, CCR5, dectin-1, NOD2 or the mannose receptor [42], [43], [44], [45], [46]. Therefore, we hoped to identify differences between Pam_3_CSK4 and *M. vaccae*-mediated effects on DC as candidate mechanisms for divergent Th polarization in our model. Comparable upregulation of CD83 and CD86 in DC primed with LPS or *M. vaccae* (100 µg/mL) and in DC primed with Pam_3_CSK4 or *M. vaccae* (10 µg/mL) did not correlate with the effects on Th2 responses. Therefore differences in DC maturation as judged by these markers are not sufficient for DC-mediated inhibition of Th2 responses. Transcriptional profiling was used to make more comprehensive comparison of DC primed with Pam_3_CSK4 or *M. vaccae*. Remarkably PCA of these data showed that the major gene expression changes induced by these stimuli were extremely similar, and markedly different to changes induced by LPS. Therefore, although *M. vaccae* is likely to stimulate multiple innate immune receptors in DC the main transcriptional responses can be mediated via TLR2, and these are not suffucient for inhibition of Th2 responses.

Transcriptional responses were mirrored by measurements of cytokines in cell culture supernatants, except for increased secretion of IL-1β in *M. vaccae* primed DC. IL-1β secretion is tightly regulated by activation of the inflammasome and caspase-1. In view of the role of this pathway as a bridge between innate and adaptive immunity [47], we considered the possibility that IL-1β is involved in inhibition of Th2 responses by *M. vaccae* primed DC, but the substantial concentrations of IL-1ra in the same samples shed doubt on the biological significance of modest increases in IL-1β. In addition, the inflammasome has been reported to augment rather than inhibit Th2 responses [48].

Analysis of transcriptional regulation in gene expression changes exclusively induced by *M. vaccae* showed striking enrichment for CREB1 binding sites and assessment of intracellular signaling pathways showed that stimulation of DC with *M. vaccae* selectively induced early activation of the CREB pathway. This finding is consistent with other reports of mycobacterial induction of CREB pathways in macrophages [49], [50] and PBMC [51], and of particular interest in the light of increasing evidence for the role of CREB in modulation of immune responses [52]. In the present study, comparable activation of signaling pathways by LPS and Pam_3_CSK4 was discordant with marked differences in transcriptional responses, cytokine production and cell surface maturation phenotype. Nonetheless, selective early activation of the CREB pathway by *M. vaccae* was reflected in the transcriptional response, suggesting that the temporal relationship or sequence of signaling events is functionally important, and supported reports that transcriptional regulation by NFκB may compete and antagonize CREB-dependent regulation [53]. However our data show that these differences in the primary (4 hour) transcriptional response to *M. vaccae* and Pam_3_CSK4, did not lead to divergence of the transcriptome at subsequent time points (24 hours). Therefore it is unlikely that the critical determinants of the immunomodulatory effects of *M. vaccae* under investigation are mediated by effects of transcription. However, further study of the cellular components upstream of CREB1 phosphorylation and the downstream consequences may provide novel insights into the molecular mechanisms of *M. vaccae* interactions with DC and DC-mediated inhibition of Th2 responses.

## Materials and Methods

### M. vaccae suspension and TLR ligands

Heat-killed *M. vaccae* (strain NCTC 11659, batch MV07) manufactured under good manufacturing practice conditions were provided by Eden Biodesign (Liverpool, U.K.). Lipopolysaccharide (LPS) from *Salmonella enterica* serotype typhimurium was obtained from Sigma Aldrich. Pam_3_CSK4, heat killed *Listeria monocytogenesis*, FSL-1, Poly(I:C), Flagellin, Gardiquimod, CL075 and OND2006 were purchased from Invivogen.

### TLR reporter cell line

Human embryonic kidney (HEK)-293 cells stably transfected with one or two TLR genes were used to screen for TLR-mediated cellular activation assessed by ELISA for IL8 production [54]. Cells expressing TLR3, TLR5, TLR7, TLR8, TLR9, TLR1/2 or TLR2/6 were obtained from InvivoGen and maintained under 10 µg/mL blasticidin selection (InvivoGen). Cells expressing CD14/TLR2 and CD14/TLR4 were a kind gift from Dr. E. Latz, (University of Massachusetts) and maintained under 5 µg/mL puromycin selection (InvivoGen).

### Primary cells

Human blood samples were obtained from healthy volunteers for isolation of peripheral blood mononuclear cells (PBMC). The study was approved by the joint University College London/University College London Hospitals National Health Service Trust Human Research Ethics Committee and written informed consent was obtained from all participants. PBMC, were prepared by density-gradient centrifugation of heparinized blood with Lymphoprep (Axis-Shield) according to the manufacturer's instructions and used to isolate CD14^+^ monocytes by magnetic cell sorting (Miltenyi Biotec). Monocytes were differentiated into dendritic cells (DC) for 4 days using GM-CSF and IL-4 as previously described [55]. T cell subsets were isolated from CD14^−ve^ PBMC using the CD4^+^ T Cell Isolation Kit II or Naive CD4^+^ T Cell Isolation Kit II respectively (Miltenyi Biotech).

### DC stimulation and co-culture with T cells

DC were stimulated for 4–24 hours with LPS, Pam_3_CSK4 or *M. vaccae*. 10 µg/mL of tetanus toxoid (National Institute for Biological Standards and Control, UK) was added for antigen specific experiments. In some experiments iDC were treated with the following inhibitors for 2 hours prior to stimulation: H89 (50 µM; Sigma-Aldrich), SQ 22536 (100 µM), PD 98059 (25 µM), SB 203580 (25 µM) and LY 294002 (25 µM) (all Calbiochem). 10^1^–10^4^ DC were mixed with either 10^5^ naïve allogeneic or unselected autologous CD4^+^ T cells in triplicate. T cell proliferation was assessed by addition of 1 µCi of [3H]thymidine for 18 hours and measurement of thymidine incorporation by liquid scintillation counting. Intracellular cytokine production by T cells was assessed on day 3, after 5 hours stimulation with 7.5 µg/mL ionomycin (Sigma Aldrich) and 125 ng/mL phorbol 12-myristate 13-acetate (Sigma Aldrich) for allogeneic cultures, or 10 µg/mL tetanus toxoid for autologous cultures in the presence of 25 µg/mL Brefeldin A. Cell surface staining of DC was performed with directly conjugated antibodies for CD83 (clone HB15e) and CD86 (clone 2331- FUN-1) (both BD Biosciences). Intracellular cytokine staining was performed with mouse anti IL-4 PE conjugated antibody (clone 8D4-8) and mouse anti IFN-γ APC conjugated antibody (clone B27) using the Cytofix/Cytoperm kit (all BD Biosciences). Intracellular FoxP3 staining was conducted on day 6 following surface staining for CD25 (clone M-A251) and CD4 (clone L200), using anti human FoxP3 antibody (clone 259D/C7) staining kit (all BD Biosciences). DC apoptosis and cell death, were detected by Annexin-V and propidium iodide (PI) staining, using the Annexin-V FITC Apoptosis Detection Kit (eBioscience). Stained cells were examined by flow cytometry using a FACScan flow cytometer (BD Biosciences). Data were analyzed with FlowJo software (Tree Star).

### Transcriptional profiling by DNA microarray

DC culture lysates collected in RLT buffer (Qiagen) were used to purify total RNA and generate Cy3 or Cy5 labelled cRNA for hybridization with Agilent 4×44K whole human genome cDNA microarrays and data acquisition as previously described [56]. Log_2_ transformed data were then subjected to LOESS normalization [57] and compared by paired T-tests (p<0.05) using MultiExperiment Viewer v4.4.1 (http://www.tm4.org/mev/). Gene lists of interest were annotated using DAVID functional annotation clustering (http://david.abcc.ncifcrf.gov), and subjected to transcription factor enrichment analysis oPOSSUM (http://www.cisreg.ca/oPOSSUM/). These analyses were restricted to genes with refseq accession numbers for which contemporary functional annotation is available. Principle component analysis was performed using the R-project (http://www.r-project.org/) to obtain a global overview of gene expression data. MIAME compliant microarray data have been submitted to the ArrayExpress database (www.ebi.ac.uk/arrayexpress), accession number: E-TABM-998.

### Quantitation of soluble factors released by DC

Cytokines and chemokines in cell culture supernatants were quantified using human Biosource multiplex bead immunoassay kits (Invitrogen) with the Luminex 200 platform LuminexIS software 2.3 (Luminex, Austin, TX).

### Assessment of cellular innate immune signaling

Cell lysates from DC cultures were collected directly into SDS sample buffer for Western blotting as previously described [56]. Rabbit anti-Ser133-phosphorylated CREB (clone 87G3; Cell Signaling Technology), rabbit anti-IκBα (clone 44D4; Cell Signaling Technology), rabbit anti-phosphorylated ERK1/2 (clone 197G2; Cell Signaling Technology), rabbit anti-phosphorylated p38 MAPK (clone 3D7; Cell Signaling Technology), and mouse anti-β-actin (Abcam) were used as primary antibodies. Classical NFκB activation was measured by quantitative confocal immunofluorescence analysis of NFκB nuclear translocation [56].
